# Key Insights From a Rapid Review of Nondental Procedural Sedation Clinical Practice Guidelines for Adults With Intellectual and Developmental Disability

**DOI:** 10.1111/jebm.70003

**Published:** 2025-02-17

**Authors:** Andrea Simpson, Jessica Smith, Matthew Yates, Hannah Barden, Cassandra Gorton, Michelle Templeton

**Affiliations:** ^1^ Centre for Developmental Disability Health Monash Health Doveton Victoria Australia; ^2^ School of Allied Health, Human Services, and Sport La Trobe University Bundoora Victoria Australia; ^3^ Faculty of Health Charles Darwin University Darwin Northern Territory Australia; ^4^ Department of Rehabilitation Flinders Medical Centre Aged & Palliative Care Flinders Drive Australia; ^5^ Brain Injury Rehabilitation Service Westmead Hospital Wentworthville New South Wales Australia; ^6^ Faculty of Medicine and Health, The University of Sydney, School of Health Sciences Camperdown New South Wales Australia; ^7^ Library Services Monash Health Clayton Victoria Australia

1

People with intellectual and developmental disabilities (IDD) experience significantly higher rates of physical and mental health conditions compared to the general population [1, 2]. This group also faces substantial barriers to accessing healthcare, often leading to unmet health needs and poor health outcomes [3]. For many people with IDD, routine clinical procedures, such as blood tests or immunizations, can be distressing [4]. This distress may stem from past trauma, complex communication needs, insufficient adjustments for sensory sensitivities, needle phobia, or the lack of accessible information about medical procedures [5]. As a result, it is not uncommon for clinicians to delay or abandon medical interventions for this population, placing individuals at risk for vaccine‐preventable diseases and delays in diagnosing or managing health conditions [6].

For this reason, people with IDD can require procedural modifications for routine medical procedures, such as blood tests and immunizations. A range of nonpharmacological interventions, such as graduated exposure [7] and distractions using auditory or visual stimuli [8], have been proposed. For individuals who experience heightened anxiety during minor procedures, pharmacological interventions like sedation may be necessary.

Clinical practice guidelines (CPGs) for sedation in this population are limited, especially outside of dental care. We carried out a rapid review aimed to identify and assess existing CPGs for procedural sedation in adults with IDD. The review followed the rapid review guidelines of the Agency for Healthcare Research and Quality [9]. A PICO framework guided the review question, focusing on CPGs for procedural sedation (excluding dental care) in adults with IDD. Electronic searches were conducted across MEDLINE, Embase, Emcare, PsycINFO, and Scopus. Study selection and data extraction were carried out by two reviewers with disagreements resolved by a third. The AGREE II tool [10] was employed to assess the quality of the included guidelines. Data were extracted from 9 studies on study characteristics, procedural models of care, and key patient safety outcomes, including procedure completion rates, adverse side effects, and escalation in sedation dosage or type.

The 9 studies originated from several countries, including the United Kingdom (*n* = 3), the United States (*n* = 5), Australia (*n* = 1), and Italy (*n* = 1). The clinical procedures across the studies ranged from premedication, behavioral strategies, desensitization programs, to virtual reality exposure therapy. Clinical qualifications varied, with most procedures involving a multidisciplinary team that included anesthetists, nurses, psychologists, behavioral therapists, and other healthcare professionals, depending on the intervention's nature. The reviewed studies revealed a mix of both pharmacological and behavioral interventions ‐ see Table 1. Out of the 9 studies, 5 reported medications as part of the clinical procedure, while the remaining 4 focused solely on behavioral or nonpharmacological interventions.

**TABLE 1:** A summary of nonpharmacological and pharmacological interventions for procedural sedation found in the study.

Intervention type	Examples	No studies	Intervention completed	Challenges
Behavioral	Desensitization programs	1	*N* = 21/24	Time‐intensive, requires multiple exposures
	Video modeling	1	*N* = 1/1	May not work for all cognitive levels
	Virtual reality exposure therapy	1	*N* = 1/1	Requires specialized equipment
	At‐home audiovisual training	1	*N* = 18/19	Compliance can be difficult and can require multiple attempts
Pharmacological	Midazolam (oral, intranasal)	2	*N* = 5/13	Risk of respiratory depression
	Topical anesthetic cream	1	Not described	May not be sufficient
	Clonidine (oral, intranasal)	1	Not described	Longer onset time
	Dexmedetomidine (intranasal)	1	Not described	Limited availability in some settings
	Naloxone (intranasal)	1	Not described	Longer onset time
	Ketamine (intranasal, IV)	1	Not described	Possible emergence reactions

Studies involving medication included the use of Midazolam, Dexmedetomidine, Clonidine, and Ketamine, delivered via various routes such as intranasal, oral, and topical [4, 11–13]. For example, Rava [4, 12] employed a combination of behavioral strategies and mild pharmacological sedation, and Tetef [13] used transmucosal sedatives. The remaining four studies [14, 15, 16, 17] focused on behavioral interventions without medication. These included desensitization programs [15], video modeling [14], at‐home audio‐visual training [17], and virtual reality exposure therapy [16]. These studies emphasized nonpharmacological approaches to managing patient anxiety or phobia. Intervention failure actions across the 9 studies generally included switching to more pharmacologically intensive strategies [4, 12] or pausing and terminating interventions for the day if signs of distress were observed, particularly in desensitization programs [15]. Of the 7 guidelines assessed for quality appraisal, 6 were recommended for use. Of these, 4 were recommended without edits [4, 12, 13, 15, 18]. One [17] was recommended with minor edits, and 1 [16] was not recommended.

One of the most striking findings was a lack of consumer involvement in the development of many existing guidelines. Consumer engagement, particularly the involvement of people with IDD and their caregivers, is crucial for creating guidelines that are both relevant and feasible in real‐world practice [19]. The absence of their input not only weakened the guidelines' foundation but also limited their potential to be effectively implemented by healthcare providers who require practical, patient‐centered recommendations.

Additionally, the variation in procedural sedation approaches across the included studies indicates a fragmented landscape of care for adults with IDD. While some guidelines incorporated pharmacological interventions such as Midazolam or Dexmedetomidine, others relied solely on behavioral strategies, creating inconsistencies in how sedation was approached in different clinical settings.

The review highlighted the scarcity of research on nondental procedural sedation in adults with IDD compared to pediatric populations. Most of the existing models of care and sedation pathways were developed with children in mind or focused solely on dental care, limiting their applicability to adult patients’ in general healthcare settings. For example, nitrous oxide, a commonly used agent for moderate to deep sedation, was notably absent from the reviewed guidelines, highlighting a significant gap given its widespread application in managing procedural distress. This omission is problematic as it overlooks a sedation option that is both effective and rapidly reversible, limiting the practical applicability of the guidelines. This variability may lead to confusion among healthcare providers and potentially impact patient safety and procedure outcomes [20].

Desensitization interventions, while potentially beneficial in reducing long‐term procedural distress for individuals with IDD, are resource‐intensive for healthcare services. These programs require significant time, specialized training, and repeated exposure sessions, involving multidisciplinary teams and often extended timelines [15]. Although successful desensitization could lead to sustained long‐term benefits, reducing the need for future sedation, its long‐term efficacy remains uncertain, and maintaining the desensitization effect may require ongoing efforts.

A critical consideration in developing procedural sedation pathways for adults with IDD is the definition and classification of sedation levels, as well as the regulations surrounding who may administer sedation and the required monitoring standards. These topics remain highly contentious due to the diverse range of sedation practices across different clinical settings and provider backgrounds. What is considered well‐established and appropriate in one context may be deemed unsuitable in another.

To ensure clarity and alignment with established frameworks, a standardized classification should be adopted.  A tiered model, which categorizes sedation based on the route of administration (e.g., oral, intranasal/inhalational, intramuscular, intravenous/anesthesia), does not fully account for dose‐dependent effects, drug combinations, or variations in sedation techniques. For instance, oral midazolam can induce mild sedation at low doses but may lead to moderate or even deep sedation at higher doses. Nitrous oxide produces moderate to deep sedation but rapidly wears off within approximately 60 seconds after discontinuation. Many procedural guidelines do not account for this type of sedation, as they assume deep sedation requires administration by an anesthetist and do not provide clear guidance on transient, reversible sedation states.

Given the authoritative nature of anesthetic guidelines, disregarding them is not a viable option. However, it is important to acknowledge that these guidelines are often developed without explicit consideration of the unique needs of individuals with IDD.  Many adopt a hospital‐centric approach that may impose unnecessary restrictions on moderate sedation in non‐hospital settings. The absence of guidance on sedation for individuals with IDD further highlights the need for supplementary protocols that bridge the gap between existing guidelines and the practical realities of procedural sedation in this population.

Future work should focus on developing tailored sedation pathways that align with best practice recommendations while addressing the specific needs of individuals with IDD. Additionally, collaboration with professional bodies and advocacy groups is essential to ensure that future revisions of sedation guidelines incorporate the perspectives and clinical experiences of those working with this population. Without such efforts, individuals with IDD may continue to face significant barriers in accessing safe and effective sedation for routine medical procedures (Table 1).

## Conflicts of Interest

The authors declare no conflicts of interest.
